# Interplay of the photon drag and the surface photogalvanic effects in the metal-semiconductor nanocomposite

**DOI:** 10.1038/s41598-018-26923-2

**Published:** 2018-06-05

**Authors:** G. M. Mikheev, A. S. Saushin, V. M. Styapshin, Yu. P. Svirko

**Affiliations:** 1 Institute of Mechanics, Udmurt Federal Research Center of the UB RAS, Izhevsk, 426067 Russia; 2 Institute of Photonics, University of Eastern Finland, Joensuu, 80101 Finland

## Abstract

Photon drag effect (PDE) and surface photogalvanic effect (SPGE) can be observed in centrosymmetric media and manifest themselves in photocurrents, the magnitude and polarity of which depend on wavevector and polarization of the excitation laser beam. PDE photocurrent originates from the transfer of the photon momentum to a free charge carrier, while SPGE photocurrent is due to diffuse scattering of the photoexcited carriers in the subsurface layer. However, despite the different underlying physical mechanisms, these photocurrents have almost indistinguishable dependencies on the polarization and the angle of incidence of the excitation laser beam. In this paper, we observe for the first time a competition between PDE and SPGE in the film containing metal (Ag-Pd) and semiconductor (PdO) nanocrystallites. We show that, depending on the angle of incidence, polarization azimuth and wavelength of the excitation laser beam, the interplay of the PDE and SPGE leads to the generation of either monopolar or bipolar nanosecond current pulses. The experiments performed allow us to visualize the contributions both these effects and obtain light-to-current conversion efficiency in a wide spectral range. Our experimental findings can be employed to control the magnitude and polarity of the light-induced current by polarization of the excitation laser beam.

## Introduction

Photon drag effect (PDE)^1,2^ and surface photogalvanic effect (SPGE)^3,4^ manifest themselves as light-induced currents, magnitude and polarity of which depend on the wavevector and polarization of the excitation laser beam. In contrast to the bulk photogalvanic effect^5^, the PDE and SPGE photocurrents can be observed in both centrosymmetric and non-centrosymmetric media^6^ including metal, semimetal and semiconducting films^7–12^, two-dimensional electron gas^13,14^, nanocarbon films^15–17^ and on the surface of bulk semiconductors and metals^3,18^.

In the visual and near-IR spectral range, the PDE is due to the transfer of the photon momentum to a charge carrier^1,2,19^, while the SPGE originates from interband transitions in the subsurface layer that produce conduction electrons with anisotropic momentum distribution. The diffuse scattering of these electrons from the surface results in the generation of the polarization-sensitive surface current^3^, which can be directed both along and perpendicular to the plane of incidence^15,17^.

The PDE and SPGE photocurrents are very sensitive to the bulk and surface electronic properties opening a way e.g. to study spin-locked surface currents in the topological insulators^20^. From the application side, the dependences of the SPGE and PDE photocurrents on the polarization and incidence angle of the excitation beam are essential e.g. for controlling the THz emission from 2D materials irradiated by femtosecond light pulses^21,22^.

In metals, the PDE and SPGE photocurrents are usually very low because they are heavily suppressed by shot cut currents. For example, in the pioneering paper^18^, the PDE and SPGE were observed in copper foil by using an ultrasensitive SQUID magnetometer at cryogenic temperature. However, if the sheet resistance *R*_*sr*_  is at the level of tens Ω/◽, the polarization-sensitive surface photocurrents generated by nanosecond laser pulses can be observed at room temperature with a broadband oscilloscope^16^. Having originated from very different mechanisms, SPGE and PDE show very similar dependences on the angle of incidence and polarization of the excitation beam^16,18,23^. This makes separating them in metals^18^ or semimetals^16,23^ a difficult experimental task.

Interplay of the PDE and SPGE can be observed in metal-semiconductor nanocomposites containing semiconductor and metal nanoparticles embedded in a dielectric matrix. In these artificial materials, irradiation with intense laser pulses can give rise to the PDE photocurrent in metal constituent, while interband transitions in semiconductor nanoparticles should produce the SPGE photocurrent. It is worth noting, that the nanocomposite should be centrosymmetric to avoid linear and circular photogalvanic effects^24–26^ and should possess relatively low conductivity to suppress shot cut currents.

One of the materials that meets these requirements is Ag/Pd nanocomposite^27^ containing semiconducting PdO and metallic Ag-Pd nanoparticles. Sheet resistance of 10–25 µm thick Ag/Pd films, which are conventionally used in electronics^27,28^, may vary in a wide range depending on the fabrication technique^28^. The band gap of PdO is not firmly established^29^ being in the range of 0.6  - 0.8 eV^30,31^, which corresponds to the 1550–2067 nm wavelength region. That is, the SPGE photocurrent can be generated under irradiation with laser pulses at wavelengths shorter than 2000 nm, while at longer wavelengths, the photocurrent will be governed by the PDE. Thus, tuning the wavelength of the excitation beam may lead to pronounced change in the magnitude and temporal evolution of the polarization-sensitive photocurrent due to interplay of the PDE and SPGE.

Very recently we have demonstrated the PDE photocurrent in the 20 μm thick Ag/Pd film with *R*_sr_ = 30 Ω/◽ irradiated with nanosecond pulses of the fundamental and second harmonic beams of the Nd^3+^:YAG laser^9,32^. The obtained conversion efficiency of about 1 nA/W is comparable with that demonstrated in the nanostructured silver and gold films^11^.

In this paper, we visualize and compare contributions of the PDE and SPGE by studying the polarization-sensitive photocurrents generated in the Ag/Pd nanocomposite films irradiated with nanosecond pulses in the wavelength range spanning from 1064 to 4000 nm. We demonstrate that the competition between PDE and SPGE manifests itself in both magnitude and temporal profile of the generated photocurrent pulse and strongly influences the power-to-current conversion efficiency. In Ag/Pd nanocomposites, unique polarization and incidence angle dependences of photocurrents are observed in a very broad spectral range that spans from IR to UV^33^. This allows one to employ this material to visualize polarization of powerful laser beams of arbitrary wavelength and/or spatial orientation of the Ag/Pd film.

## Results and Discussion

**Figure 1:**
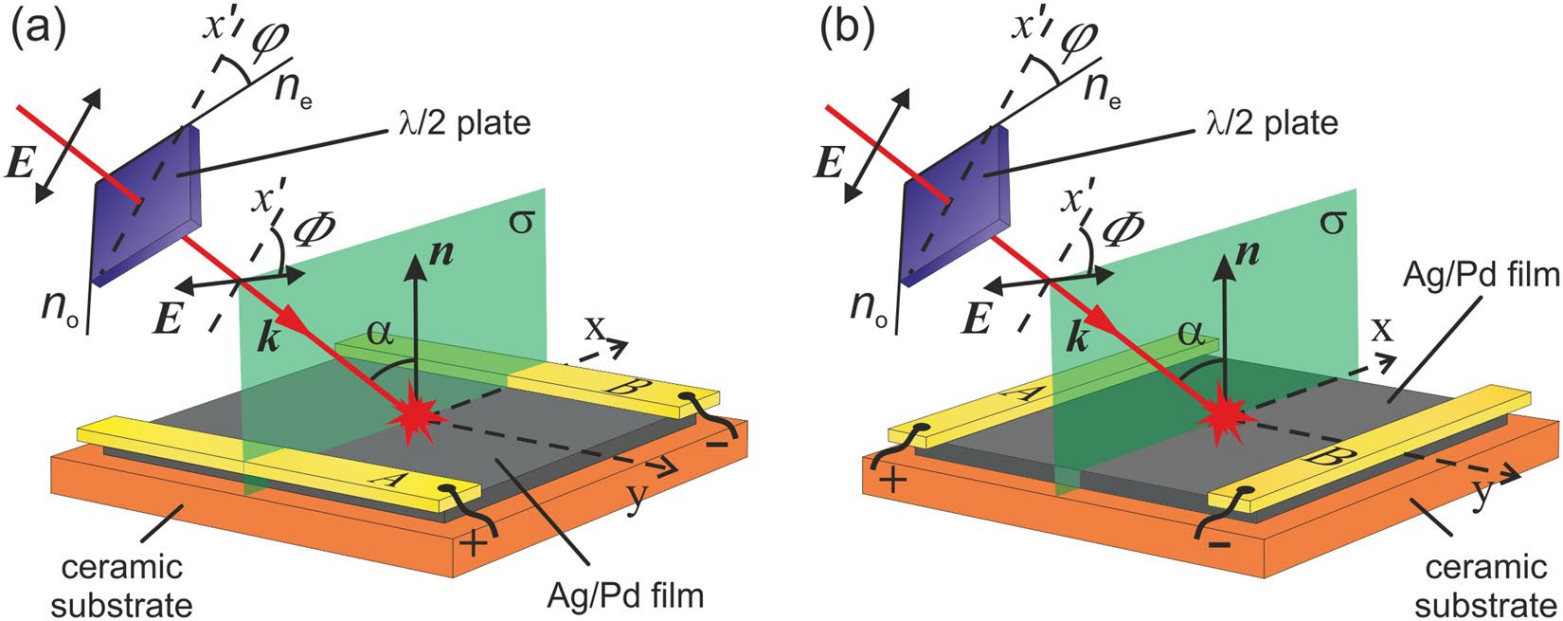
Sketch of the experimental setup for registration of longitudinal (**a**) and transverse (**b**) photocurrents in Ag/Pd nanocomposite film. Electrodes *A* and *B* are deposited on the sample’s edges perpendicular (**a**) and parallel (**b**) to the plane of incidence σ, which coincides with the (*xz*) plane of the laboratory Cartesian frame. The rotation of the λ/2 plate by the angle φ changes the polarization azimuth angle Φ = 2φ of the incident beam; α is the angle of incidence, ***k*** is wavevector, ***n*** is the unit vector of the normal to the film.

In the experiment, we measure voltage across the electrodes attached to the 20 µm thick Ag/Pd film (see Methods), which is irradiated with nanosecond laser pulses. The laser beam, which is linearly polarized along the *x*′ axis in the plane of incidence σ (see Fig. 1), passes through a half-wave plate and hits the film at the incidence angle α. We control the polarization azimuth Φ of the incident beam by rotating the half-wave plate by the angle φ = Φ/2. One can observe from Fig. 1 that at φ = 0 and φ = 45*°*, the incident beam is *p*-polarized (Φ = 0) and *s*-polarized (Φ = 90°), respectively.

**Figure 2:**
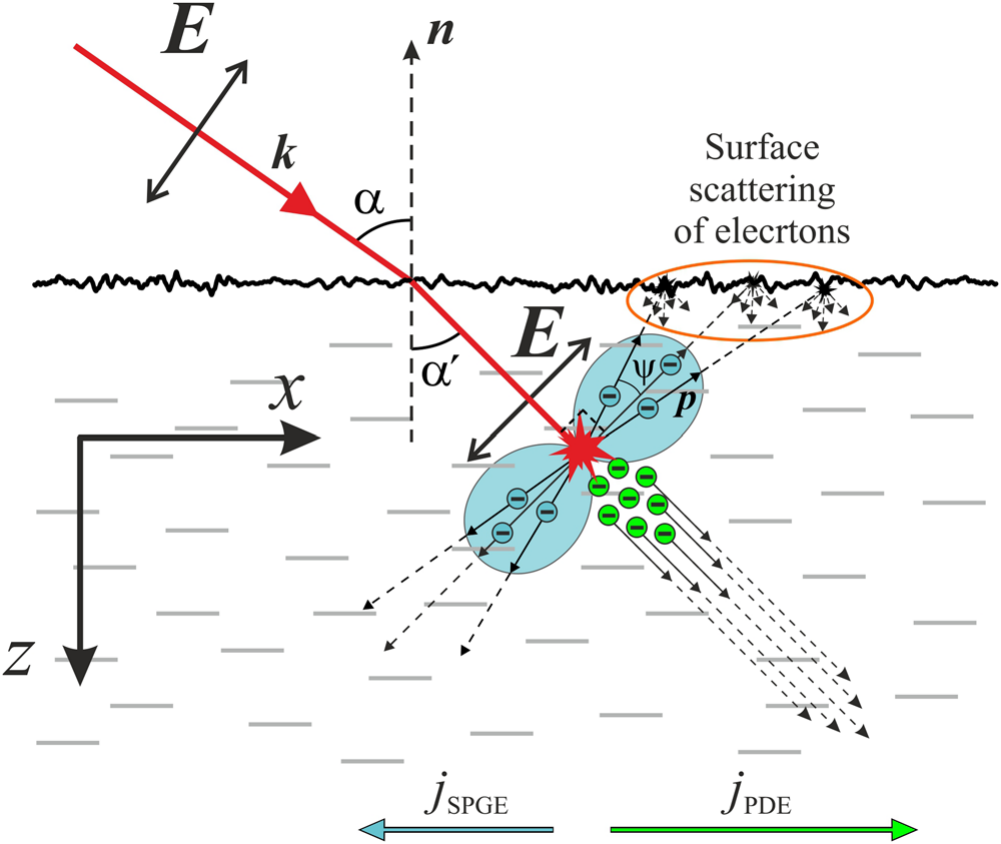
Schematic illustration of the SPGE and PDE photocurrents excited by *p*-polarized laser beam in the subsurface layer of Ag/Pd nanocomposite. The probability of the interband transition in semiconductor PdO nanoparticles is proportional to cos^2^ψ, where ψ is the angle between the electric field ***E*** of a light and the electron quasimomentum ***p****.* That is, the same amount of the free electrons moving toward the surface and away from the surface will be created. The diffuse scattering from the surface results in the imbalance between the number of electrons moving along +*x* and −*x* axes giving rise to the SPGE current *j*_*x*,*SPGE*_ along −*x* axis. The longitudinal PDE photocurrent *j*_*x*,*PDE*_ along +*x* axis arises due to photon momenta transfer to the free electrons in the metallic constituent of the nanocomposite.

The PDE and SPGE photocurrents, which are generated in the irradiated film, have opposite polarities. This can be understood qualitatively by considering interaction of the *p*-polarized laser beam with the nanocomposite constituents, i.e. with metallic Ag-Pd and semiconducting PdO nanoparticles. When a conduction electron in the Ag-Pd solid solution absorbs photon, the photon momentum is transferred to the electron, which starts moving along the beam direction^19^ (see green circles in Fig. 2), giving rise to the longitudinal PDE photocurrent. The magnitude of the PDE current density along +*x* axis (see Fig. 2) can be estimated as^34^:

1 $${j}_{x,PDE}\propto \beta I\frac{e{\tau }_{e}}{mc}\,\sin \,\alpha \,\cos \,\alpha ,$$

where *β* is the absorption coefficient of the film, *I* is the beam intensity, *e*, *m* and *τ*_*e*_ are electron charge, mass and collision time, respectively, *c* is speed of light, and α is the angle of incidence.

The SPGE current originates from the photogeneration of the conduction electrons due to interband transition in the PdO nanocrystals situated in the subsurface area. The probability of the interband transition is proportional to (***Ep***)^2^ ∝ cos^2^*ψ* (blue shapes in Fig. 2), where *ψ* is the angle between the electron quasimomentum ***p*** and electric field of the light wave ***E***^3,4,18^. Since for *p*-polarized beam ***E*** lies in the *xz*-plane (see Fig. 2), the probability of the interband transition is the same for electrons moving towards and away from the nanocomposite surface. That is, the number of conduction electrons generated by light and moving, for example, along the +*x* axis and towards the surface (Fig. 2) would be equal to the number of electrons moving along *−x* and away from the surface. In the bulk nanocomposite, photogenerated electrons of both groups are scattered on the lattice defects and phonons. However, in the subsurface layer of thickness less than the electron mean free path, the electrons of the first group experience an additional scattering from the surface. If the surface scattering is diffuse, i.e. if the *x*-component of the electron momentum changes after reflection from the surface, electrons traveling to the right in Fig. 2 will lose their momentum more rapidly than the electrons traveling to the left, because the latter are scattered only in the interior. Such an imbalance results in a net electron flux towards −*x* axis. It is worth noting that for an ideal surface, the net electron flux is zero because photogenerated electrons preserve *x*-component of the momentum after specular reflection. Correspondingly, the SPGE surface current density *g*_*x,SPGE*_ can be estimated from^3^:

2 $${g}_{x,SPGE}\propto P\frac{eI}{\hslash \omega }{\rm{\Lambda }}\,\sin \,\alpha \,\cos \,\alpha $$

where 0 ≤ *P* ≤ 1 (*P* = 0 and *P* = 1 correspond to the specular and diffuse electron scattering, respectively), Λ is the electron mean free path, *ħω* is photon energy. The SPGE photocurrent disappears when the electrons are mirror reflected from the semiconductor surface and when the incident beam is *s*-polarized.

One can observe from Fig. 2 that net electron currents generated in the subsurface area due to SPGE and PDE propagate in the −*x* and +*x* directions, respectively. Therefore, when the Ag/Pd film is irradiated with a laser pulse, the longitudinal PDE and SPGE currents manifest themselves as positive and negative photovoltage, respectively, generated between electrodes *A* and *B* shown in Fig. 1.

It is worth noting that since the electron mean free pass in the Ag/Pd composite is longer than the light penetration depth^35^, the PDE surface photocurrent also has a transverse component^4^, i.e. conduction electrons in the irradiated subsurface area can also move perpendicular to the plane of incidence (see Fig. 1b). The transverse SPGE photocurrent is also allowed if the electric field ***E*** of the excitation beam does not lie in the plane of incidence, i.e. when both *x*- and *y*-components of the electric field in Fig. 1 are nonzero. In this case the interband transition probability, which is determined by (***Ep***)^2^, includes a term proportional to $2Re\{{E}_{x}^{\ast }{E}_{y}^{\ast }{p}_{x}{p}_{y}\}$, where subscripts label Cartesian axes (see Figs 1b and 2). This term permits interband transitions only for electrons having nonzero both *x*- and *y*-components of the quasimomentum. That is at Φ *≠* 0 and Φ *≠* 90°, the longitudinal and transverse SPGE currents take place simultaneously. It is worth noting that the transverse SPGE and PDE photocurrents vanish for *p*- and *s*-polarized excitation beams, while the longitudinal PDE photocurrent is non-zero for any polarization.

**Figure 3:**
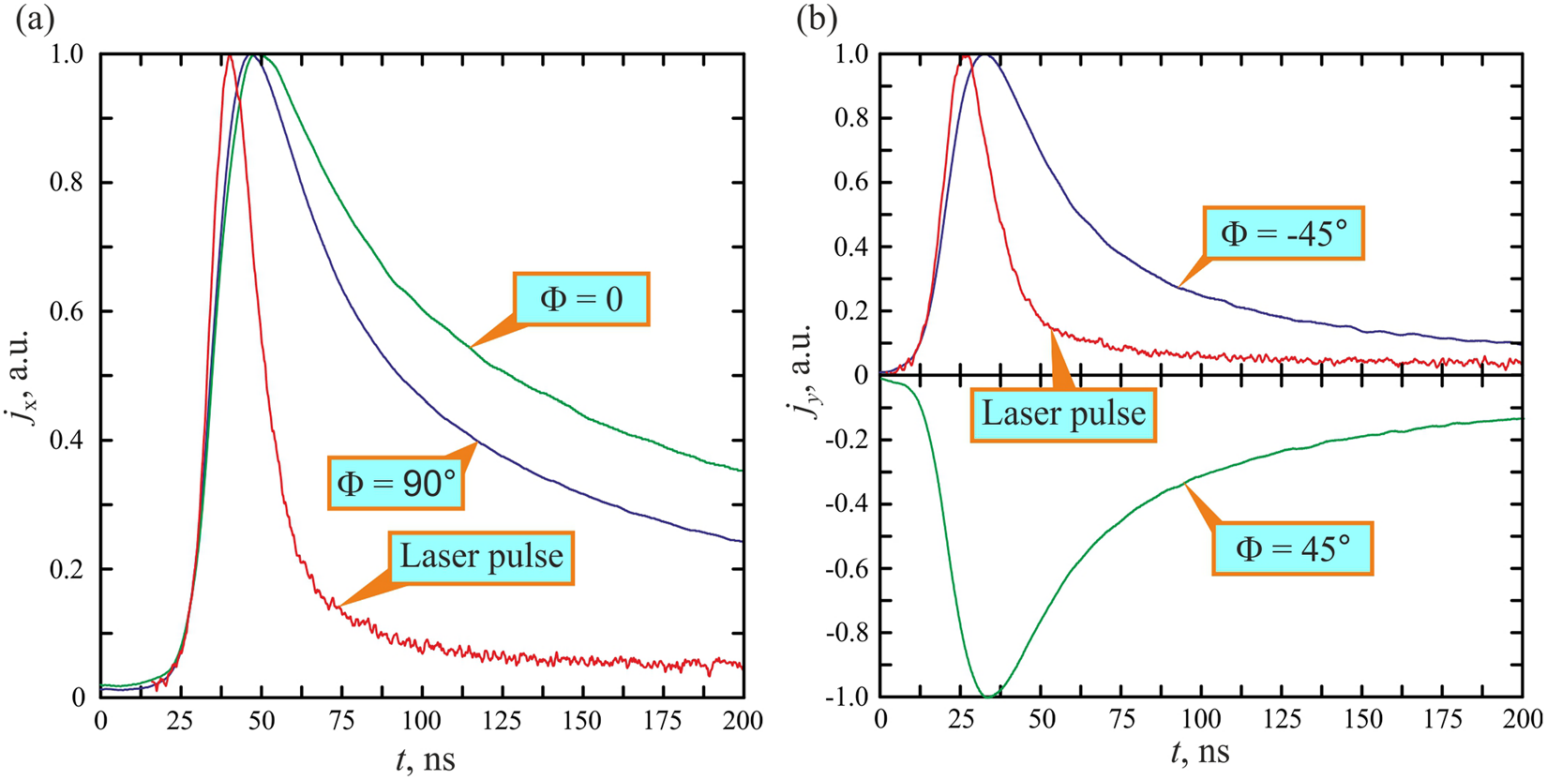
Oscillograms of the longitudinal (**a**) and transverse (**b**) photocurrent pulses at the excitation beam polarization azimuths of Φ = 0, ±45°, 90° and α = 45°. Orange line shows temporal profile of the excitation laser pulse at the wavelength of 1064 nm.

Figure 3a shows that at the incidence angle of α = 45°, *p*- and *s*-polarized 1064 nm laser beams induce longitudinal photocurrents of the same polarity, however the pulse generated by the *p*-polarized laser beam is longer than that generated by the *s*-polarized beam. The transverse photocurrent polarity (Fig. 3b) reverses when the polarization azimuth of the excitation beam swaps from Φ = 45° to Φ = −45°.

**Figure 4:**
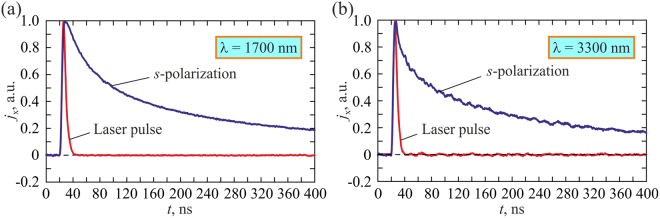
Oscillograms of the longitudinal photocurrent pulses induced by *s*-polarized laser beam at 1700 (**a**) and 3300 (**b**) nm.

**Figure 5:**
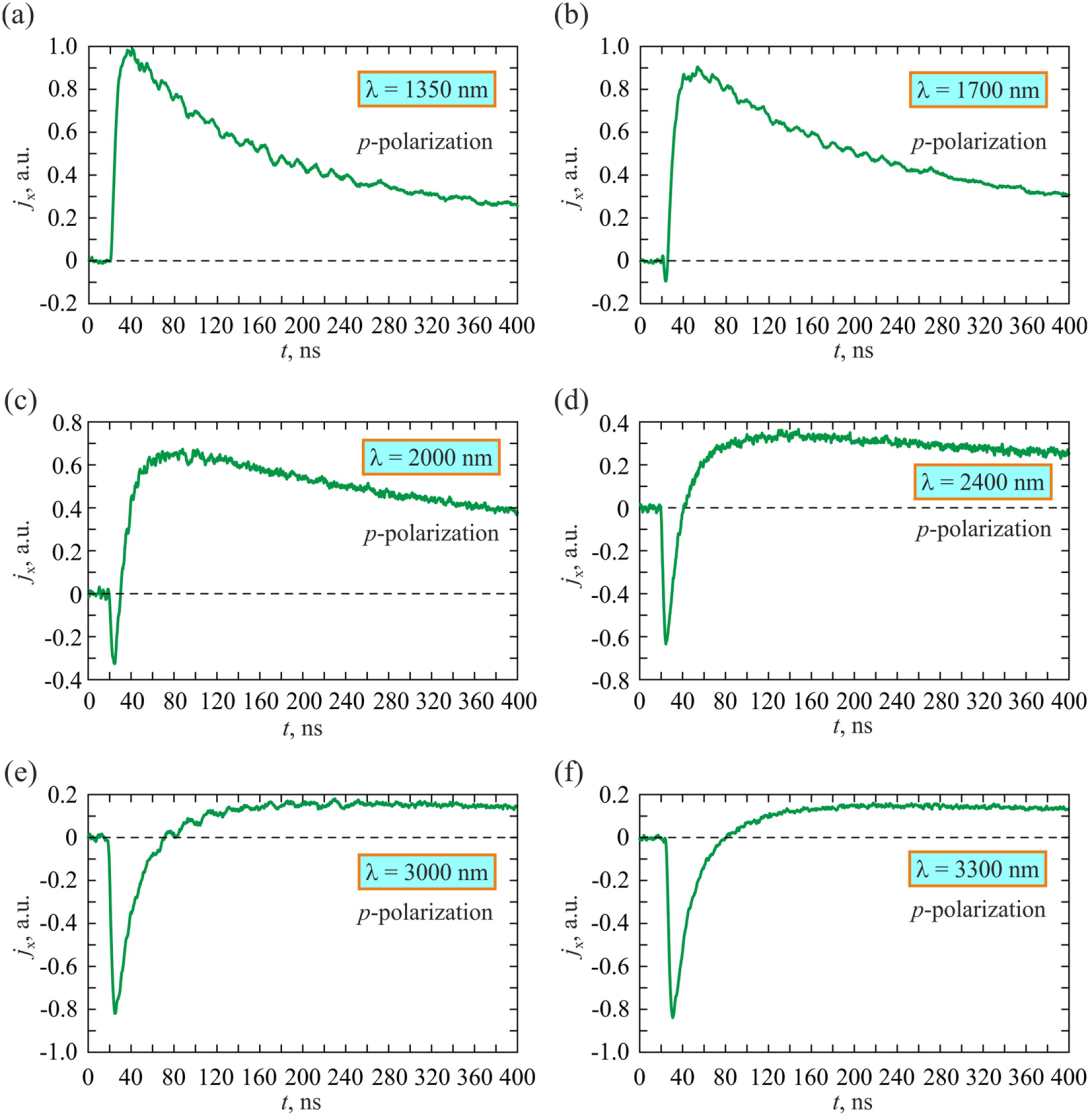
Oscillograms of longitudinal photocurrent pulses produced by the *p*-polarized laser beam at 1350 (**a**), 1700 (**b**), 2000 (**c**), 2400 (**d**), 3000 (**e**) and 3300 (f) nm.

By performing measurements in the wavelength range of 1350–4000 nm we found that the *s*-polarized excitation beam produces unipolar longitudinal photocurrent pulse, which shape is virtually independent of the wavelength (see Fig. 4). In contrast, the temporal profile of the longitudinal photocurrent *j*_x_(*t*) produced by the *p-*polarized laser beam essentially depends on the pump wavelength (see Fig. 5). Specifically, if the excitation wavelength is shorter than 1670 nm, *j*_*x*_(*t*) is a unipolar pulse, which is longer than that produced by the *s-*polarized beam. However, at the excitation wavelength of 1670 nm, a negative pulse emerges at the leading edge of the positive longitudinal photocurrent. The amplitude and duration of this negative pulse grows as the excitation wavelength increases. One can observe from Fig. 5c that at the excitation wavelength of 2000 nm, the longitudinal photocurrent is transformed into a distinct bipolar pulse with a sharp negative front and a long positive tail. This is because of simultaneous generation of the PDE and SPGE photocurrents, which have opposite polarities and different durations as well as different rise and fall times. The temporal profile of the resulting pulse is determined by relative magnitudes of *j*_*PDE*_ and *j*_*SPGE*_. When the wavelength of the *p*-polarized excitation beam is shorter than 1670 nm (see Figs 3a and 5a) the longitudinal photocurrent pulse remains unipolar indicating that the PDE prevails. Since at the *s*-polarized excitation beam the SPGE current vanishes, the longitudinal photocurrent is a positive unipolar pulse irrespective of the wavelength.

**Figure 6:**
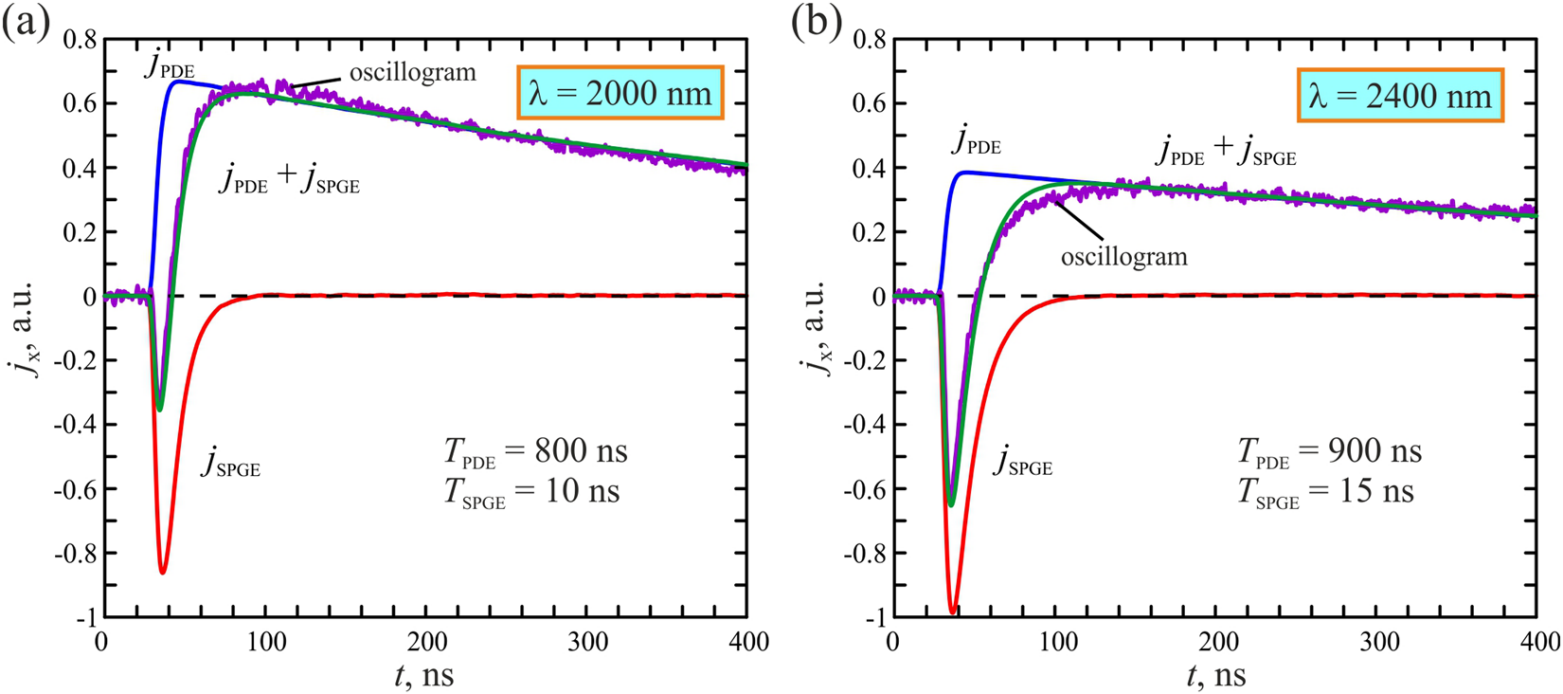
Temporal profiles of the measured photocurrent (violet) produced by *p*-polarized laser pulses at the wavelengths of 2000 nm (**a**) and 2400 nm (**b**). Blue, red and green lines show results of fitting of the PDE, SPGE and total photocurrent pulses, respectively, using Eq. (3).

Since both PDE and SPGE are second order nonlinear effects^36^, the temporal evolution of the PDE and SPGE photocurrents can be described by the following equation:

3 $${j}_{PDE,SPGE}(t)={f}_{PDE,SPGE}({\rm{\Phi }},\alpha ){\int }_{0}^{\infty }I(t-\tau )\exp [-\frac{\tau }{{T}_{PDE,SPGE}}]d\tau ,$$

where *I*(*t*) is the excitation pulse intensity, *f*_*PDE*_ (Φ, α) and *f*_*SPGE*_ (Φ, α) have opposite signs and describe polarization and incidence angle dependences of the PDE and SPGE, respectively, *T*_*PDE*_ and *T*_*SPGE*_ are response times of the relevant mechanisms. Temporal profiles of the PDE and SPGE photocurrents at the excitation wavelengths of 2000 nm (Fig. 6a) and 2400 nm (Fig. 6b) were obtained by using Eq. (3). One can observe that for both PDE and SPGE, the response times at the excitation wavelength of 2000 nm are slightly shorter than those at the excitation wavelength of 2400 nm. Such a shortening of the response times may originate from the composition of the nanocomposite because at the excitation wavelength of 2400 nm, the interband transition takes place in a smaller number of PdO nanoparticles than that at the excitation wavelength of 2000 nm. However, detailed description of the observed dependence on the wavelength needs further study.

**Figure 7:**
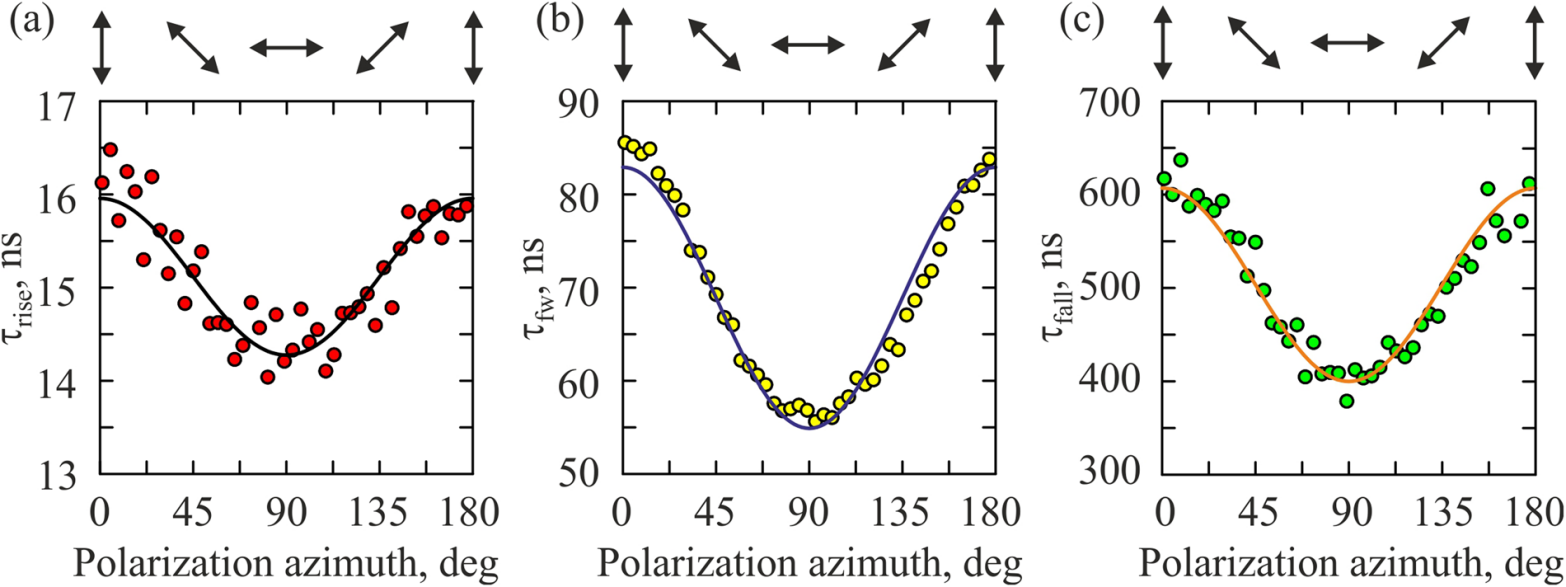
The rise time (**a**), pulse duration (**b**) and fall time (**c**) as functions of the polarization azimuth Φ of the excitation beam. Orientations of the electric field for different Φ are shown at the top.

In practice, it is convenient to describe the temporal profile of the photocurrent in terms of the rise time τ_rise_, pulse duration τ_hw_ and fall time τ_fall_ (see Methods). Figure 7 demonstrates that these parameters for the longitudinal photocurrent depend on the polarization of the excitation beam. Specifically, the rise time, pulse duration, and fall time of the longitudinal photocurrent for the *p*-polarized excitation beam are longer than those for the *s*-polarized one. Dependence of τ_rise_, τ_hw_ and τ_fall_ on Φ is well described by the following equation:

4 $${\tau }_{M}({\rm{\Phi }})={\tau }_{M}({\rm{\Phi }}=0){\cos }^{2}{\rm{\Phi }}+{\tau }_{M}({\rm{\Phi }}=\pi /2){\sin }^{2}{\rm{\Phi }},$$

where subscript *M* labels *rise, hw* and *fall*.

There is a common belief that temporal profile of PDE photocurrent reproduces the shape of the nanosecond excitation pulse^17,37–39^ because of the subpicosecond carrier momentum relaxation time in metals. However, in the Ag/Pd nanocomposite,τ_rise_, τ_hw_ and τ_fall_ are longer than the corresponding temporal characteristics of the excitation pulse (see Figs 3 and 7). Such a slowing down of the response is due to the presence of the Schottky barriers between metallic Ag-Pd and semiconductor PdO nanocrystallites^35^.

**Figure 8:**
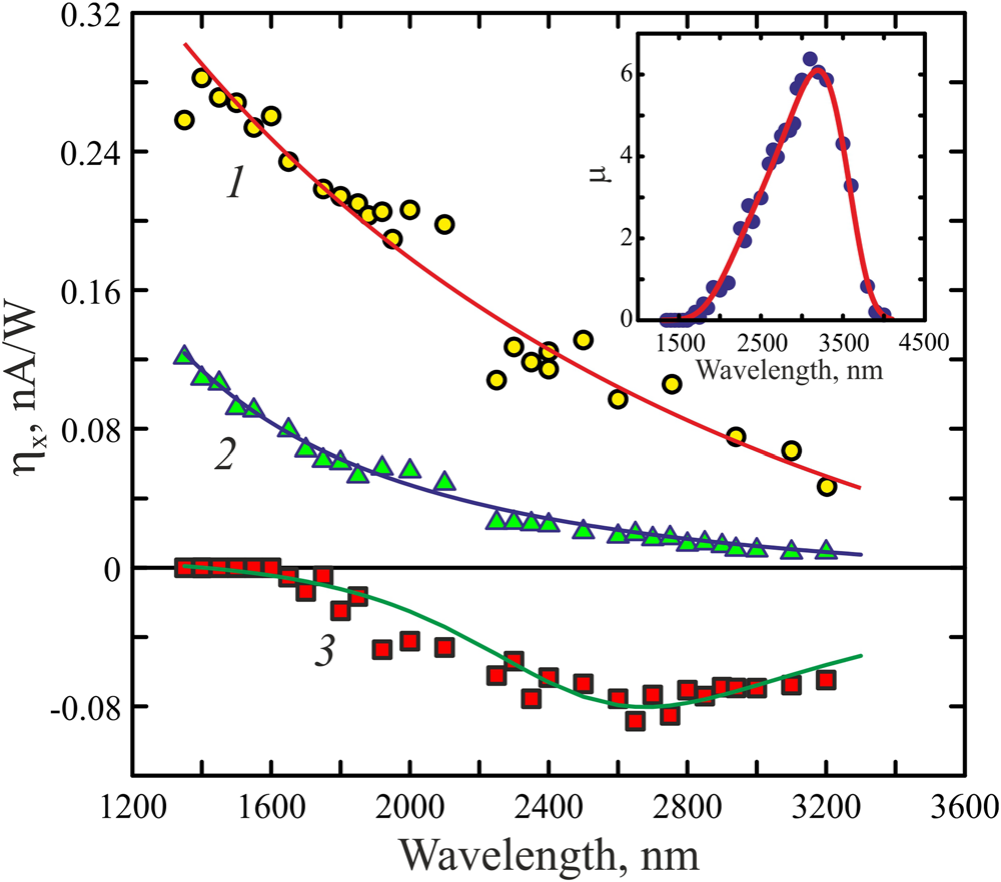
Conversion efficiency as a function of the excitation wavelength. (1) *s*-polarized excitation beam; (2) *p*-polarized excitation beam (positive pulse); (3) *p*-polarized excitation beam (negative pulse). Solid lines are guides to the eye. Inset shows the wavelength dependence of the ratio of the negative and positive pulses produced by *p*-polarized excitation beam.

It is convenient to describe the wavelength dependence of the photocurrent in terms of the longitudinal (η_x_ = *j*_x_τ_p_/*E*_in_) and transversal (η_*y*_ = *j*_y_τ_p_/*E*_in_) conversion efficiencies, where τ_p_ and *E*_in_ are the duration and energy of the excitation laser pulse, respectively. In order to describe the bipolar current pulse generated by the *p-*polarized laser beam, we introduce conversion efficiencies for the positive (η_x,p,pos_) and negative (η_x,p,neg_) parts of the pulse. One can observe from Fig. 8 that the longitudinal conversion efficiency η_x,s_ (λ) for the *s*-polarized excitation beam and η_x,p,pos_ (λ) both decrease monotonously when the excitation wavelength increases. This is because the longer the wavelength, the smaller photon momentum to be transferred to electrons and the higher the reflectivity of the Ag/Pd nanocomposite films. Both factors lead to decrease of the PDE current when excitation wavelength increases. It is worth noting that similar dependence has been obtained for the Ag/Pd nanocomposite in visual spectral range^9^.

However, the inset in Fig. 8 shows that the ratio of the amplitudes of the negative and positive parts of the bipolar current pulse, μ = *|*η_x, p, neg_ *|*/η_x, p, pos_, is non-monotonous function of the wavelength. Specifically, μ(λ) maximum at λ = 3350 nm and shows sharp decrease in the wavelength range of 3400–3800 nm. Thus, the negative pulse at the leading edge of the photocurrent vanishes at wavelength of 4000 nm, i.e. at the *p*-polarized excitation, the bipolar photocurrent pulse exists in the wavelength range of 1670–4000 nm.

The observed non-monotonous dependence of the negative pulse amplitude on the excitation wavelength can be explained by the fact that in the Ag/Pd nanocomposite, the SPGE is due to interband transitions in PdO nanocrystallites. In bulk PdO, the band gap lies in the range from 0.6 to 0.8 eV that corresponds to the wavelength region of 1550–2067 nm^30,31^, however, in the nanocomposite, the band gap also depends on the size of the nanoparticles and dielectric properties of the host^40–42^. In the Ag/Pd nanocomposite, the diameter of the PdO nanoparticles, which are surrounded by Ag-Pd nanocrystallites, ranges from 28 nm to hundreds of nanometers. As a result, the band gap of the nanocomposite may vary in a wide range, being substantially different from that of bulk PdO. That is why we observe negative photovoltage when the excitation pulse has a wavelength of about 2000 nm, however it vanishes at longer wavelengths (e.g. 4000 nm, see the inset to Fig. 8). Thus, in our experimental conditions, the negative current pulse (see inset in Fig. 8) vanishes at excitation wavelength of about 4000 nm indicating that the film contains no PdO nanocrystallites with band gap smaller than 0.3 eV.

**Figure 9:**
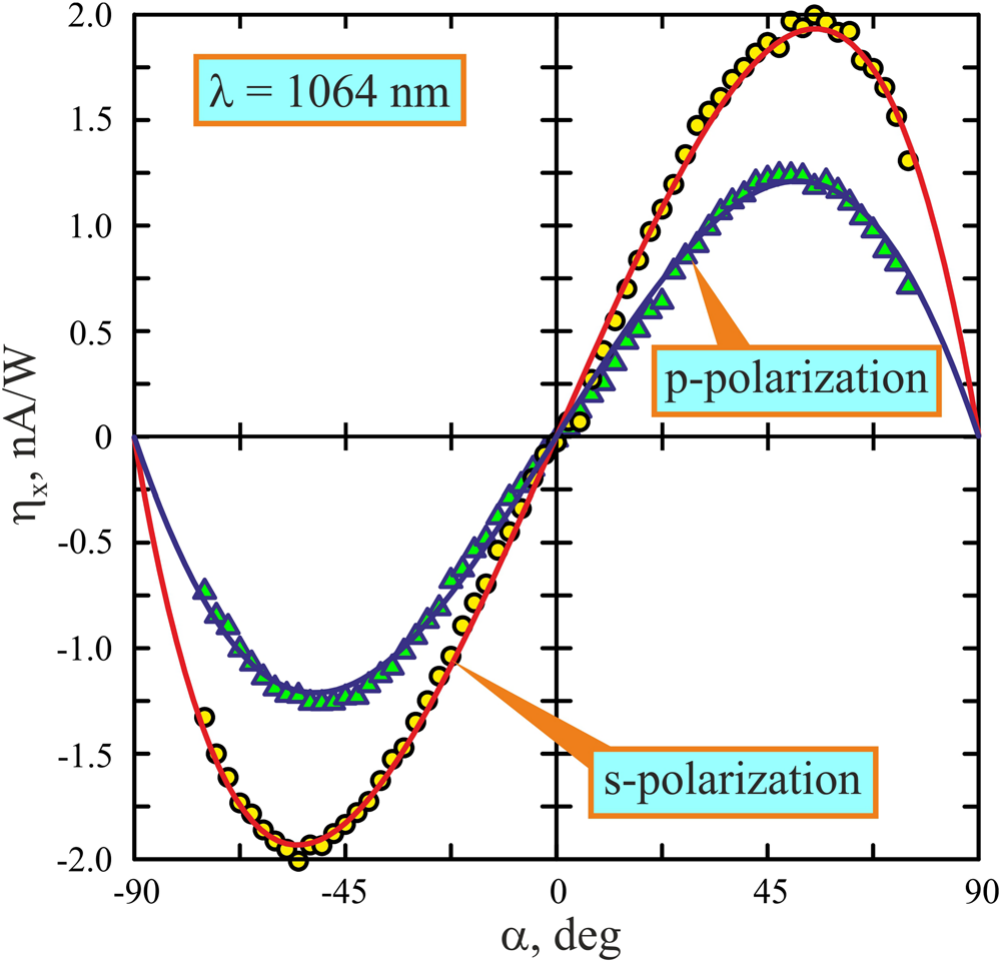
Incidence angle dependence of the longitudinal conversion efficiency (ηx) for *p*polarized (triangles) and *s*-polarized (circles) excitation beams at λ = 1064 nm. The blue and orange solid lines show results of fitting with η_x,p_ = 1.86sin(2α)/[0.36cos(α) + 1]^2^ and η_x, s_ = 3.68sin(2α)/[0.61cos(α) + 1]^2^, respectively.

Figure 9 shows longitudinal conversion efficiency η_x_ = *j*_x_τ_p_/*E*_in_ as a function of the incidence angle α for the *p*- and *s*-polarizations. One can see that longitudinal photocurrent reverses polarity for mirrored incidence, being an odd function of the incidence angle, η_x_(−α) = −η_x_(α), and vanishing at normal incidence (α = 0). It is worth noting that at the excitation wavelength of 1064 nm, the longitudinal conversion efficiency for *s*-polarized beam is higher than that for the *p*-polarized beam.

**Figure 10:**
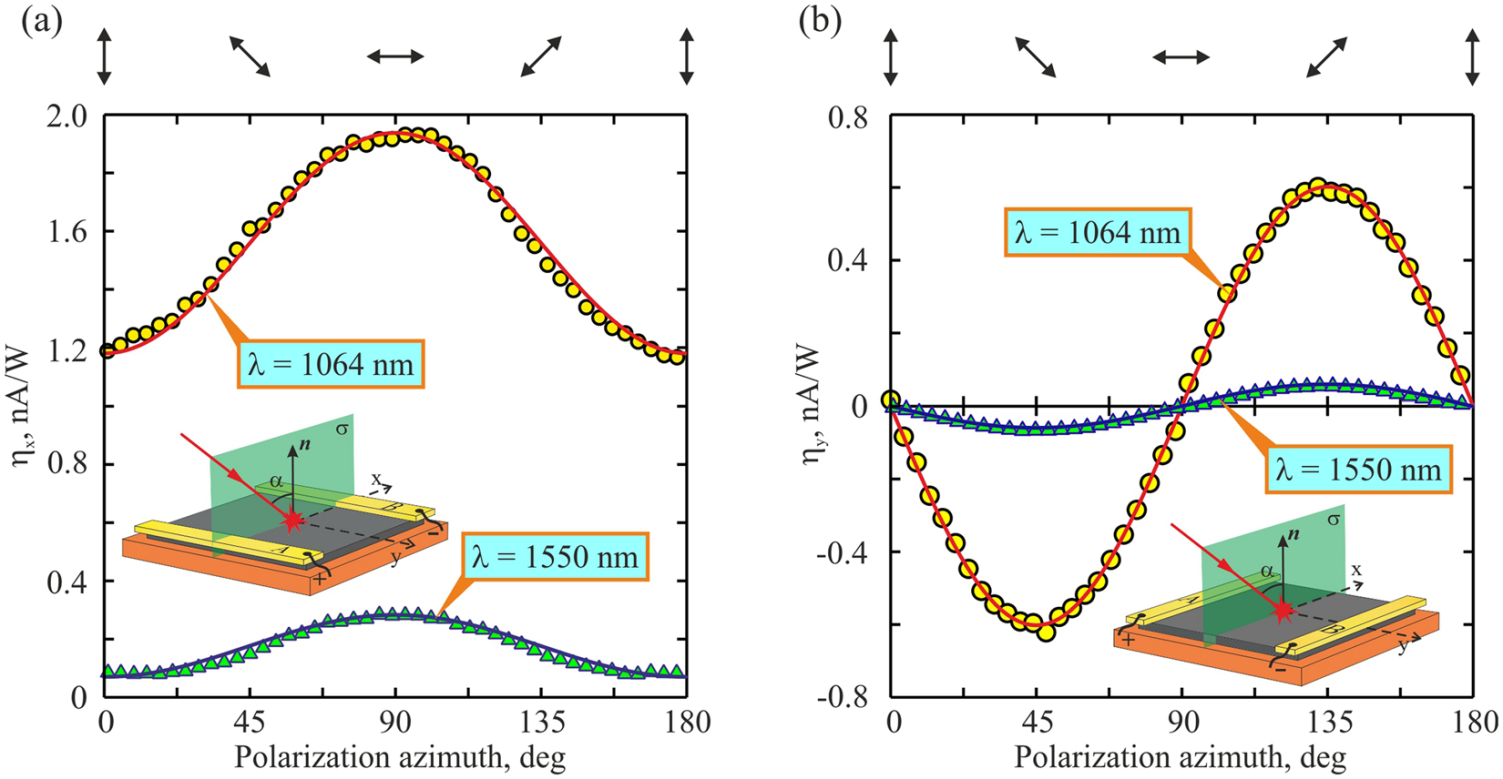
Dependences of the conversion efficiency on the excitation beam polarization azimuth Φ for the longitudinal (**a**) and transverse (**b**) photocurrents at λ = 1064 (yellow circles) and 1550 nm (green triangles). Orange solid lines show fitting with η_x_ (λ = 1064 nm) = 1.19·cos^2^(Φ) + 1.94·sin^2^(Φ) and η_y_ (λ = 1064 nm) = −0.6sin(2Φ). Blue solid lines show fitting with η_x_ (λ = 1550 nm) = 0.093 cos^2^(Φ) + 0.295·sin^2^(Φ) and η_y_ (λ = 1550 nm) = −0.059sin(2Φ). Orientations of the electric field for different Φ are shown at the top.

The obtained dependence of the longitudinal photocurrent on the angle of incidence α shown in Fig. 9 is typical for the PDE^7,10,11,16,17^. Specifically, the PDE photocurrent has opposite polarities for mirrored incidence angles and vanishes at α = 0. However, our experimental results can not be explained in terms of the PDE only. This is because the photon drag current is proportional to the number of the absorbed photons^1^, i.e. the higher the absorption coefficient of the medium, the stronger the PDE current. Since the absorption losses in metals for the *p*-polarized beam are higher than those for the *s*-polarized one (see, for example^43^), the PDE current conversion efficiency in Ag/Pd film for the *p-*polarized excitation beam should be higher than that for the s-polarized one. Since in the experiment (see Fig. 9) we observed the bigger conversion efficiency for the *s*-polarized excitation beam, one may expect that the SPGE also contributes to the photocurrent. Figure 10 presents the experimentally obtained dependences of conversion efficiencies for the longitudinal (η_x_ = *j*_x_τ_p_/*E*_in_) and transverse (η_y_ = *j*_y_τ_p_/*E*_in_) photocurrents on the polarization azimuth Φ of the incident beam at α = 45°. One can observe from Fig. 10 that current conversion efficiencies at excitation wavelengths of 1064 nm and 1550 nm can be approximated as

5 $${\eta }_{x}({\rm{\Phi }})={\eta }_{x}({\rm{\Phi }}=0){\cos }^{2}{\rm{\Phi }}+{\eta }_{x}({\rm{\Phi }}=\pi /2){\sin }^{2}{\rm{\Phi }},$$

6 $${\eta }_{y}({\rm{\Phi }})={\eta }_{y}({\rm{\Phi }}=\pi /4)\sin \,2{\rm{\Phi }}.$$

Thus, our experimental results show that η_x_ and η_y_ are even and odd functions Φ, respectively.

In an isotropic medium, the polarization azimuth dependence of the SPGE longitudinal photocurrent can be described by the following equation^3,18^:

7 $${j}_{x,SPGE}({\rm{\Phi }})={j}_{x,SPGE}({\rm{\Phi }}=0){\cos }^{2}{\rm{\Phi }}.$$

One can observe from Eq. (7) that only PDE contributes to the photocurrent at the *s*-polarized excitation beam (Φ = 90°), while at the *p*-polarized excitation (Φ = 0°) both PDE and SPGE contribute to the photocurrent. However, since the longitudinal photocurrent remains positive for all polarization azimuths of the excitation beam (see Fig. 10a), one may conclude that at the excitation wavelengths of 1064 nm and 1550 nm, the PDE dominates the photocurrent in the Ag/Pd film. At the same time, at longer wavelengths, the SPGE prevails for the *p*-polarized excitation beam (see Fig. 5d–f). Decrease of the polarization azimuth from Φ = 90° down to Φ = 0° results in the increase of the negative longitudinal SPGE photocurrent, and hence in the suppressing of the amplitude and change of the temporal characteristics of the photocurrent pulse, which eventually becomes bipolar.

## Conclusion

We demonstrate that the measurement of the photoexcited currents in the metal-semiconductor nanocomposite allows us to visualize the interplay of the SPGE and PDE. This opens avenues towards the study of the interface’s influence on carriers’ dynamics in the subsurface layer. In the wavelength range of 1700–3350 nm, the ratio of amplitudes of negative and positive parts of the bipolar current pulse for the *p*-polarized excitation beam in Ag/Pg nanocomposite is uniquely determined by the excitation wavelength. This experimental finding provides an opportunity to visualize the excitation wavelength without using a spectrum analyzer, i.e. by non-optical means. We also show that it is possible to control the magnitude and polarity of the light-induced current by changing the polarization of excitation laser beam. Since the Ag/Pd films, which can sustain high temperatures and irradiation with intense laser pulses, are capable of generating a photocurrent depending on polarization and angle of incidence of excitation laser beam, they can be employed as a position and orientation sensors.

## Methods

The Ag/Pd samples were fabricated by using the thick-film technology, which is conventionally used to produce hybrid integrated circuits and other electronic devices^27,28^. This technology is based on the thermal processing of a paste prepared by mixing the metal and ceramic powders with an organic vehicle allowing one to obtain nanocomposites with prescribed electronic properties. Ag/Pd nanocomposite contains Ag-Pd solid solution, palladium oxide (PdO) and silver oxide (Ag_2_O) nanoparticles, glass microparticles, and organic vehicle^27^. Since the maximum temperature of the baking process was as high as *T*_bur_ = 878 K^32^, glass microparticles melted forming a quasi-uniform distribution of metal and semiconductor nanoparticles in the film. The fabricated films have lateral size of 20 × 20 mm^2^ and thickness of 20 μm. Our measurements showed that the films possess *p*-type conductivity of 15.2 Ω^−1^ cm^−1^ at the hole concentration of 9.2 × 10^20^ cm^−3^ and mobility of 1 × 10^−1^ cm^2^/(V × s). X-ray diffraction data reveal that the Ag/Pd films contain nanocrystals of Ag-Pd solid solution (80.3 wt%), palladium oxide PdO (18.7 wt%), and silver oxide Ag_2_O (1 wt%)^32^. It was found that the Ag-Pd solid solution with about 26% Pd content has a fcc lattice with lattice parameter *a* = 0.4036 nm, while PdO has a centrosymmetric tetragonal lattice PdO belonging to the spatial symmetry group ${D}_{4h}^{9}$^29,44^ with lattice parameters *a* = 0.3043 nm and *c* = 0.5337 nm. The measurement of the *X*-ray diffraction line width showed that the minimum crystallites size of Ag-Pd and PdO are 39 and 28 nm, respectively^32^.

In contrast to black phosphorous^45^ and other 2D materials, which have recently attracted a widespread attention for their photonic and optoelectronic applications^46^, Ag/Pd nanocomposites have a linear volt-ampere characteristic, while their electric and photovoltaic properties remain the same in a wide temperature range both in vacuum and air atmosphere. They also sustain irradiation with intense laser pulses. The nanocomposite may have impurities, however since their concentration is rather low, they do not produce a sizable effect on their electronic properties, which are determined by the Ag-Pd solid solution and PdO^32,47^.

**Figure 11:**
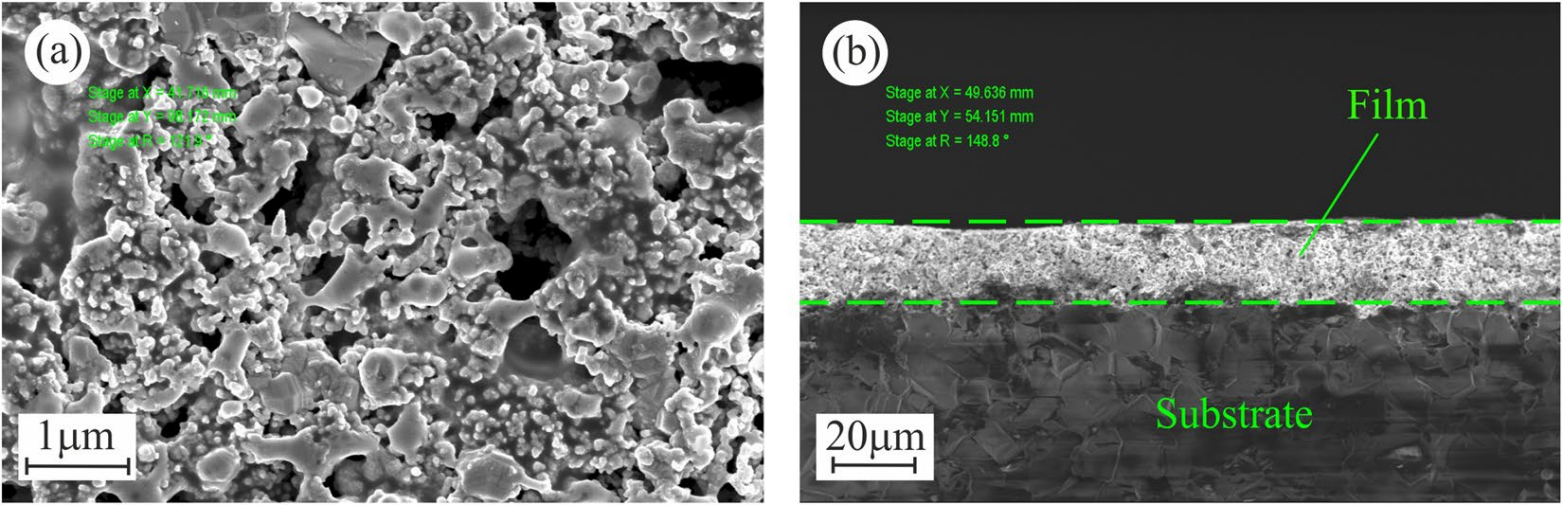
Scanning electron microscope images of the surface (**a**) and the cross-section (**b**) of the Ag/Pd nanocomposite film.

The SEM image in Fig. 11 shows that the fabricated Ag/Pd nanocomposite is a porous film with thickness of about 20 μm and pore size ranging from 25 to 500 nm. Since the pores radii are smaller than excitation wavelengths, we believe that the porosity of the material does not affect the obtained dependences of the photoexcited currents on the incidence angle and the polarization of the excitation beam.

In the experiment, we measured the voltage between electrodes *A* and *B* attached to the film irradiated with nanosecond laser pulses (see Fig. 1). The dc resistance between electrodes was 29 Ω. The electrodes were connected to a digital oscilloscope with input resistance of *r* = 50 Ω via a coaxial cable. The sketch of the experimental setup is shown in Fig. 1. We studied photovoltages *U*_x_ and *U*_y_ arising between electrodes *A* and *B* when plane of incidence was perpendicular (Fig. 1a) and parallel (Fig. 1b) to the electrodes, respectively. A special care was taken to avoid irradiation of the electrodes. The longitudinal (Fig. 1a) and transverse (Fig. 1b) – with respect to the plane of incidence σ – photocurrents were defined as *j*_x_ = *U*_x_/*r* and *j*_y_ = *U*_y_/*r*, repectively. The temporal evolution of the photocurrent pulses was characterized by measurement of the rise time τ_rise_ and fall time τ_fall_, which were defined with respect to the 0.1 and 0.9 of the pulse amplitude. The duration of the photocurrent pulse τ_hw_ was defined with respect to the 0.5 of the maximum.

We measured photoresponse of the Ag/Pd nanocomposite in the spectral range of 1064–4000 nm by using a *Q*-switched single-mode Nd^3+^:YAG laser (repetition rate 1 Hz, the full width at half maximum (FWHM) was 19 ns) and the Laser Vision^TM^ optical parametric generator and amplifier (1350–5000 nm, 10 Hz repetition rate, FWHM varied from 6 to 8 ns depending on the wavelength). The energy of the incident pulses *E*_in_ was measured using pyroelectric energy detector QE25 (Gentec-EO). The temporal profile of the Nd^3+^:YAG laser pulses was revealed by the high-speed photodetector (Thorlabs SIR5-FC; rise and fall time <70 ps) and broadband oscilloscope (Tektronix TDS7704). The linear polarization of the optical parametric generator radiation pulses was provided by a stack of silicon wafers placed at the Brewster angle, while their temporal characteristics were measured using a high-speed IR photodetector (Vigo-System Ltd, PD-10.6-3; response time <1 ns) and a digital oscilloscope (LeCroy 42Xs) with a bandwidth of 400 MHz. In order to improve the signal-to-noise ratio in the conversion efficiency measurements, each experimental point was obtained by averaging over 30 laser pulses. The investigation of the temporal evolution of the photocurrent was performed by averaging over 100 pulses. In order to suppress the systematic errors, we measured energy of each excitation pulse by a calibrated photodiode. In addition, in order to obtain the dependence of the conversion efficiency on the angle of incidence and polarization azimuth, we used the ratio of the current averaged over 30 laser pulses to the pulse energy, which was also averaged over 30 laser pulses.
